# The longitudinal connection between depressive symptoms and inflammation: Mediation by sleep quality

**DOI:** 10.1371/journal.pone.0269033

**Published:** 2022-05-26

**Authors:** Sunmi Song, Natasha N. DeMeo, David M. Almeida, Marzieh Majd, Christopher G. Engeland, Jennifer E. Graham-Engeland

**Affiliations:** 1 Department of Health Sciences, Graduate School, Korea University, Seoul, Republic of Korea; 2 Department of Biobehavioral Health, The Pennsylvania State University, University Park, PA, United States of America; 3 Department of Human Development and Family Studies, The Pennsylvania State University, University Park, PA, United States of America; 4 Department of Psychological Sciences, Rice University, Houston, TX, United States of America; 5 The College of Nursing, The Pennsylvania State University, University Park, PA, United States of America; University of Colorado Denver, UNITED STATES

## Abstract

Although there is a strong association between depressive symptoms and markers of inflammation, it remains unclear whether depressive symptoms at one point in life may predict inflammation later in life. Moreover, despite extant literature linking sleep with both depressive symptoms and inflammation, there is little research investigating poor sleep as a mechanism linking depressive symptoms with later inflammation. The links between depression and physical health can also vary by gender. In longitudinal analyses with data from the Midlife in the United States (MIDUS) study, we examined whether depressive symptoms were associated with inflammatory markers 11 years later and whether these associations were mediated by sleep disturbances or moderated by gender. Participants reported depressive symptoms and demographic information at baseline. At 11-year follow-up, the same participants (*n* = 968) reported depressive symptoms, sleep quality and duration using validated scale items, and provided a blood sample from which inflammatory markers interleukin-6 (IL-6) and C-reactive protein (CRP) were quantified. Actigraphy assessment of sleep was obtained in a subsample (*n* = 276). After adjusting for concurrent depressive symptoms and other relevant covariates, baseline depressive symptoms were associated with CRP 11 years later in the full sample, and with IL-6 among women. Subjective sleep quality mediated the association between depressive symptoms and CRP. Results suggest that depressive symptoms may be longitudinally associated with inflammation; however, directionality issues cannot be determined from the present work, particularly as inflammation markers (which might have been associated with baseline depressive symptoms) were not available at baseline. Findings further suggest that longitudinal associations between depressive symptoms and inflammation may potentially be explained by sleep and may reflect gender specific patterns.

## Introduction

Depressive disorders are increasing worldwide: From 2005 to 2015, the number of clinically depressed individuals increased by 18.4% to an estimated 311 million [1]. Major depressive disorder often coincides with chronic diseases such as cardiovascular disease, arthritis, and diabetes [2,3], many of which are leading causes of death and disability [4]. Subclinical levels of depressive symptoms are also longitudinally associated with cardiac disease morbidity and mortality [5,6] above and beyond the effect of clinical diagnosis of a depressive disorder [7]. Despite this health burden, our understanding of factors that connect depressive symptoms and physical health is limited. In recent years, low-grade systemic inflammation has emerged as a potential pathway linking depressive symptoms to many chronic diseases [8,9]. Over time, sustained systemic inflammation can promote the development and instability of arterial plaques [10] and is associated with higher risk for hypertension, cardiovascular disease, and numerous other chronic diseases later in life [11]. Biomarkers of peripheral inflammation, such as inflammatory cytokines and C-reactive protein (CRP), are useful indicators of current and future health and (like depression) have been linked with poor sleep [8,12,13]. One possibility that has been insufficiently investigated is that a depressive episode at one point in life may be linked with inflammation later in life [14–17], and that poor sleep is a potential mechanism of such an association [18,19].

### Depression and inflammation

The association between depression and inflammation appears to be bi-directional [8,20]. Both clinical and animal studies have demonstrated that inflammation can induce depressive symptoms. Further, it is well documented that increases in systemic inflammation via inflammatory challenge (e.g., endotoxin exposure) leads to sickness behavior [21], which encompasses many of the same symptoms of depression, albeit acutely. Most prospective studies have found that higher systemic inflammation at baseline was associated longitudinally with higher levels of depression-related outcomes [22–24]. A number of biological mechanisms have been posited to explain how inflammation affects the course of depression via neural, humoral, and cellular pathways [For review see, 8,9,24].

Several studies also support the prospect that depression may be associated with future inflammation, although this evidence is less conclusive. For example, studies using both experimental models and clinical trials suggest that anti-depressants, such as serotonin reuptake inhibitors, can reduce endotoxin-induced inflammation in healthy adults and circulating levels of cytokines in clinically depressed adults [25,26]. Prospective cohort studies that have examined whether depression is associated longitudinally with inflammation have provided mixed findings. For instance, a cohort study of participants from ages 9 to 21 reported that both depression (obtained one to two years earlier than CRP assessments) and cumulative depressive episodes were longitudinally associated with CRP, even after controlling for a range of relevant covariates (including gender, age, race, low socioeconomic status [SES], body mass index [BMI], alcohol or other drug use, and medication use [27]. Similarly, in a study of older adults, depression six years earlier was associated with levels of interleukin-6 (IL-6) and (marginally) CRP [28]. However, a study of midlife adults reported that depression was associated with CRP five years later only among Black participants [29], and four years later only among men [30]. Two other studies reported no associations between depressive symptoms and CRP and/or IL-6 over as many as 12 years [23,31].

Both theory and literature that are focused on physiological responses to stress support the premise that depressive symptoms may have long-term lingering health consequences, including later life inflammation and inflammatory-related disease. The “scar hypothesis” of depression [32] posits that a depressive episode can leave lasting changes in self-concept that lead to vulnerability in mood disturbances and exaggerated stress reactivity. In support of this theory, depression appears to be linked with exaggerated inflammatory responses to stress, perhaps via cross-sensitization between stress and cytokine reactivity [8]. For example, depressive symptoms were associated with greater increases in IL-6 in response to a laboratory stressor in adults [33]. Further, in comparison to non-depressed individuals, those who are clinically depressed show greater inflammatory reactivity to laboratory stressors [34]. Inflammatory responses to acute stress are less likely to attenuate after repeated exposure compared to the rapid habituation typically observed in cortisol responses [35,36]; as such, lack of habituation to stress may be one of the pathways by which depression leads to heightened stress reactivity and higher inflammation over time. In line with this assumption, formerly depressed adults were more likely to report more major and minor daily stressors and a steeper decline in positive mood on a stressful day than those without history of depression [37]. Moreover, affect and affective responses to stress are also linked with inflammation [38–40]. Together, such findings support the possibility that depression can have a long-lasting impact in ways that contribute to higher inflammation.

With regard to gender, both epidemiological and experimental studies have indicated gender differences in the linkage between depression and inflammation [17]. However, the majority of studies have not examined gender as a moderator of this linkage but rather have just controlled for it [41]. Several explanations have been suggested for observed gender differences in the link between depression and inflammation, including differences in neural activity following inflammatory responses in men and women [42], sex hormones [43], and differences in rates of several factors associated with inflammation in men and women, including obesity, physical inactivity, and childhood adversity [17]. Differences in inflammatory signaling and variation in inflammatory reactivity to stress by sex/gender may also help explain women’s higher susceptibility to depression compared to men [41,44]. For example, women were more likely than men to show depressive symptoms in response to in vivo inflammatory challenge [44]. In summary, the literature remains inconclusive about whether levels of depressive symptoms are associated with later inflammation, over what time period, and how this association might differ by gender.

### Sleep as a potential behavioral mechanism

One key behavioral factor that may help explain prospective linkages between depressive symptoms and inflammation is sleep. Sleep disturbance is a common symptom of depression. Altered sleep patterns, including early morning wakening or difficulty falling asleep (i.e., increased sleep latency), abnormal (short or long) sleep duration, as well as insomnia symptoms have been reported in as many as 83% of depressed patients [45]. Sleep disturbances are often clustered in a subgroup of depressive symptoms known as “somatic symptoms”, which includes sleep problems, fatigue, and changes in appetite. Some evidence suggests that sleep disturbances in depressed individuals may contribute to higher levels of inflammation. For example, compared to matched controls, depressed individuals appeared to have higher nighttime levels of IL-6 which were explained by sleep latency and rapid eye movement (REM) density [46]. In addition, somatic symptoms of depression (e.g., sleep disturbance) have been associated with elevated inflammatory markers beyond both mood and cognitive symptoms and such symptoms are associated with higher inflammation over time [41]. However, previous studies support the possibility that sleep disturbances may have a unique influence on inflammation from depressive mood. In a meta-analysis of cross-sectional studies examining non-depressed participants, sleep disturbances (poor sleep quality, long sleep duration [> 8 hours], and insomnia complaints) were associated with higher levels of both CRP and IL-6 [47], suggesting that various indicators of sleep disturbances can be associated with higher levels of inflammation in the absence of comorbid depression. Together, these findings support the need to examine whether sleep disturbances may be one of the important factors that help link depressive symptoms with higher levels of inflammatory markers later in life.

### Purpose of the study

The present study utilized data from the Midlife in the United States (MIDUS) study to examine the association between depressive symptoms at baseline and markers of inflammation (CRP, IL-6) 11 years later in a non-clinical sample of middle aged and older adults, and whether such associations were mediated by two primary indicators of sleep disturbances (self-reported overall sleep quality and actigraphy-based sleep efficiency). We hypothesized that depressive symptoms at baseline would be associated with higher levels of both CRP and IL-6 after adjusting for concurrent depression and demographic and physical health related covariates; further, we expected that lower sleep quality and actigraphy-assessed sleep efficiency would at least partially mediate the longitudinal association between depressive symptoms and inflammation. We also examined the mediating role of sleep duration, specifically. Although sleep duration was included as one component in the overall sleep quality measure used in this study, the mediating role of sleep duration was further examined separately (as a sensitivity analysis) to verify whether the effect of sleep quality was driven by abnormal sleep duration. Lastly, on an exploratory basis (due to mixed findings in the extant literature) we examined potential moderation by gender of the association between depressive symptoms and inflammatory markers, and potential mediation by our indicators of sleep disturbances.

## Methods

**Fig 1:**
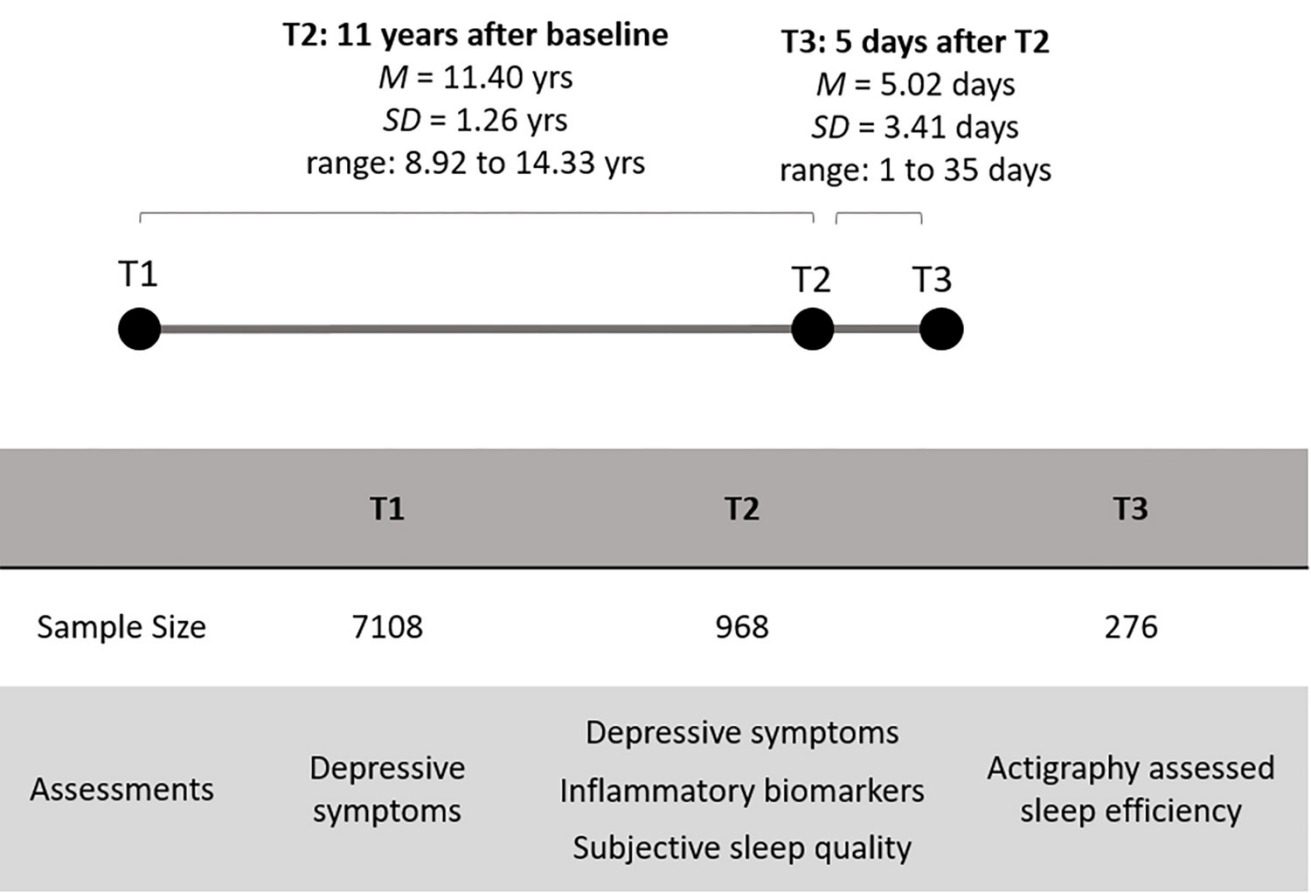
Timeline, sample size, and assessments of MIDUS data related to the present research.

The current research used data from the Midlife in the United States (MIDUS) study, which is comprised of a national probability sample of noninstitutionalized, English-speaking midlife adults. A summary of the assessment timeline and sample details of the MIDUS study are shown in Fig 1. Self-reported depressive symptoms were measured at baseline in “MIDUS 1” (T1), along with demographic information. Individuals who also participated in “MIDUS 2 Biological Project” at 11 years after the baseline (T2) took part in an overnight stay at one of three General Clinical Research Centers (GCRCs), which included self-reported measures of depressive symptoms, sleep quality and duration and a fasting blood draw (later used to assess inflammatory markers). Finally, participants from one of the three GCRCs also provided continuous actigraphy data at home for seven days at 5 days (on average) after T2 (T3), which provided assessment of sleep efficiency. MIDUS data are publicly available through the ICPSR data repository (https://www.icpsr.umich.edu) and a more detailed description of the subsamples and overall study procedures are provided elsewhere [48,49]. The study was approved by the institutional review board at each participating center, and informed written consent was obtained from all participants.

Data from 7108 participants were collected at T1, with 1255 of those participants also completing the GCRC visit at T2. Data from 287 participants were excluded due to missing data for depressive symptoms, inflammatory markers, or demographic or health covariates. The present study thus utilized a resulting sample of 968 participants (mean age = 46.26, *SD* = 11.75; 54.2% women) to examine the association between T1 depressive symptoms on inflammatory markers at T2. For sleep mediation models, a subset of participants were missing values for either overall sleep quality (*n* = 91) or sleep duration specifically (*n* = 3) and were excluded from the respective mediation analyses. Finally, the measurement of sleep efficiency excluded those (*n* = 692) who were not part of the actigraphy data collection phase. We examined whether key sample characteristics of the resulting T2 and T3 samples (which were used for current analyses) varied from the baseline T1 sample. The T2 sample was not significantly different in levels of depressive symptoms (*p* = .51) or having at least one chronic health condition at baseline (*p* = .13), relative to the baseline T1 sample. Similarly, the T3 subsample (who participated in actigraphy) did not differ significantly in having any chronic health condition (*p* = .15) or having depressive symptoms at T1 (*p* = .59). Further, levels of CRP and IL-6 were not significantly different between the T3 subsample and the T2 sample (*p* = .35 for CRP; *p* = .38 for IL-6).

### Measures

#### Depressive symptoms

Depressive symptoms were measured by the World Health Organization’s Composite International Diagnostic Interview Short [CIDI-SF; 50] via phone interviews at T1. Interviewers asked whether the participant had felt “sad, blue, or depressed” for two weeks or more in a row in the past 12 months. A response of “yes” led subsequently to 7 questions related to the experience of specific depressive symptoms, including 1) losing interest in most things, 2) losing appetite, 3) feeling more tired than usual, 4) trouble concentrating, 5) feeling down on oneself, 6) trouble sleeping, and 7) having suicidal thoughts, which were coded 1 = “yes”, 0 = “no”; total possible range of 0 to 7 with ≥ 4 subsequent symptoms indicating clinical depression [51]. Psychometric studies have shown good agreement between the CIDI-SF classifications and diagnoses for major depressive disorders made via the structured clinical interview for the Diagnostic and Statistical Manual of Mental Disorders (DSM)-5 [52]. Cronbach’s α for the CIDI-SF depressive symptom subscale was .85 for the present sample. In addition, depressive symptoms at T2 that were measured by the 20-item Center for Epidemiological Studies-Depression (CES-D) scale [53] were utilized to control for concurrent depressive symptoms. The CES-D asks about the frequency of various symptoms over the last week. Each item is rated on a 4-point scale, ranged from ‘0 = rarely or none of the time’ to ‘3 = most or all of the time’. Total scores range from 0 to 60 with ≥ 16 indicating those at risk for clinical depression.

#### Subjective sleep quality and sleep duration

Subjective sleep quality was assessed during the clinical stay (T2) via the Pittsburgh Sleep Quality Index [PSQI; 54]. The PSQI consists of 19 items and measures seven components of sleep during the past month. The total PSQI score was used to indicate level of overall subjective sleep quality. Sleep duration (used in sensitivity analyses separately) was measured by an item in the scale assessing “hours of actual sleep at night”. Sleep duration data were classified into 0 = average sleep (7–9 hours), 1 = short sleep (< 7 hours), and 2 = long sleep (> 9 hours) for the analysis in accordance with previous research and national sleep guidelines [47,55]. The scale generally has good internal consistency, test-retest reliability, and agreement with clinical diagnosis for sleep disorders [54]. The internal consistency of the PSQI in the present sample was *α* = .75.

#### Objective sleep efficiency

Participants wore a provided Mini-Mitter actiwatch-64 activity monitor (Philips Corporation, Andover, MA) and kept a sleep diary for seven consecutive days at T3. This was scheduled to begin the Tuesday morning following the clinical stay at T2 and ended upon waking the following Tuesday [56]. The actiwatch detected the number of movements every 30-second-epoch and categorized each epoch as wake or sleep; each epoch was classified as “sleep” if the total activity count was less than or equal to the wake threshold value of 40 and if the epoch was during a potential sleep period based on the daily sleep diary. Participants were also asked to report any information related to incomplete data (e.g., forgot to wear actiwatch) or unexpected experiences related to sleep (e.g., travel to different time zone), which were reviewed in data cleaning procedures. Finally, daily sleep efficiency was computed via established methods (Total number of epochs that were scored as sleep / Total number of epochs between the start and end of bedtime) × 100] [57] and averaged across the seven days.

#### Covariates

In all models, depressive symptoms at T2 were used as a covariate to examine the unique effects of depressive symptoms at T1. Based on known associations with depressive symptoms and inflammation in the literature [58], age, gender, education, race, household income at T1 were utilized as demographic covariates. To control for other factors that relate to inflammation and that may be confounded with depressive symptoms, body mass index (BMI), smoking status, chronic health conditions, and medication use at T2 were utilized as health covariates. Smoking status was coded as 0 = No smoking, 1 = Current or past smoking. A variable representing chronic health conditions was dummy coded as 0 (no condition reported) or 1 (at least one condition reported over the past 12 months [e.g., asthma, autoimmune disorders, etc.]). Two categories of medications (blood pressure medications and medications to control cholesterol [e.g., statins]) were coded as 0 (“no”) and 1 (“yes”), respectively.

### Biochemical analyses

In MIDUS, both CRP and IL-6 were measured from fasting blood samples that were collected at 7am at the end of the clinical stay. Samples were processed using standard procedures, stored at -60 to -80°C at each collection location, and shipped monthly to the MIDUS Biocore Lab, where they were stored at -65°C until assayed.

#### Serum IL-6 and CRP

Serum IL-6 was assayed using a commercially available high-sensitivity enzyme-linked immunosorbent assay (Quantikine, R&D Systems, Minneapolis, MN), with a lower limit of detection of 0.16 pg/mL. Samples were tested in duplicate and values that varied by more than 5% between duplicates were subject to repeat testing. The laboratory intra-assay coefficient of variance (CV) was 4.1%, and the inter-assay CV was 12.9%. The serum levels of CRP at T2 were measured by a particle-enhanced immunonephelometric assay (Macy, Hayes, & Tracy, 1997). The intra-assay CV was 2.3 to 4.4%, and the inter-assay CV ranged from 2.1 to 5.7%. As CRP levels over 10 mg/L may indicate the presence of acute infection [59], individuals with CRP levels ≥ 10 mg/L (*n* = 54) were removed. In later sensitivity analyses, we determined that the significance or magnitude of associations in results were not affected by excluding individuals with CRP ≥ 10 mg/L.

### Statistical analysis

A natural log transformation was applied to each inflammatory marker to normalize distributions. Quintile divisions of household income were used for analyses. Descriptive statistics and gender differences in key study variables were analyzed using *t*-tests for continuous and normally distributed variables (age, BMI, the PSQI sleep quality, sleep duration and log transformed CRP and IL-6), Chi-square tests for categorical variables (gender, education, race, smoking status, depression status, medication use variables, chronic disease status), and the Mann-Whitney tests for income, depressive symptoms, and sleep efficiency due to their skewed distributions. Pairwise deletion was used for correlations and listwise deletion for calculating means, standard deviations, and percentages. Pearson correlations were reported for the associations among study variables, with the exception of the sleep duration variable. Due to the unique inverted U shape of associations between sleep duration categories of less than 7hrs (short sleep), 7-9hrs (adequate sleep), and over 9hrs (long sleep) and health outcomes, ANOVA with posthoc comparisons were used to examine whether levels of key study variables differed by sleep duration categories. Descriptive statistics were conducted using IBM SPSS 25.0 (Armonk, NY: IBM Corp).

Multiple regression and path analyses were conducted using Mplus 7.3 (Muthén & Muthén, 1998–2014) in accordance with recommendations for both main effects and mediation models [60]. All models were estimated with the Maximum Likelihood estimator and bootstrapped 1000 times to obtain a 95% bias corrected confidence interval for the beta coefficients, with the exception of sleep duration. In sensitivity analyses with sleep duration, the theta parameterization (instead of the default delta) was applied with mean-and-variance-adjusted weighted least squares (WLSMV) estimator and bootstrapping because sleep duration was a categorical variable [61]. When the bootstrapped confidence interval and *p*-value for judging significance did not agree, we took the bootstrapped confidence interval over the *p*-value, which is robust against non-symmetric or non-normal distributions of the statistical outputs. The main effect model was tested with three different sets of covariates in addition to T2 depressive symptoms: 1) Model 1 with all examined demographic covariates at T1, 2) Model 2 with all examined health covariates at T2, and 3) our final model with all demographic and health covariates. For the mediation effect models, only significant or marginally significant covariates from the final models were included in order to achieve parsimonious models with sufficient statistical power to test mediational pathways.

Moderation by gender was examined for significant associations by using multi-group analysis. The analysis utilized a Chi-square difference test, which compares a model with constraints for coefficients for the depressive symptoms on inflammatory markers for women and men to be equal with a model without such constraints. As an additional sensitivity analysis, the potential influence of women’s menopausal status or use of hormonal therapy was examined in models with significant gender moderation.

## Results

### Description of the sample

Table 1 presents descriptive characteristics of the current study sample with data for inflammatory markers (*n* = 968). The sample included 54.2% women and an average age of 46.26 years (*SD* = 11.75) that ranged from 25 to 74 years old at baseline. Median household income was $66,250. The sample was well educated, with 72.8% having some college or higher education and included 94.2% White participants and 2.7% African-American/Black participants. The rate of any experience or treatment for emotional disorders (including depression, anxiety or others) at T1 was 20.25%. The proportion of those classified to be at risk for clinical depression was 13.7% at T1 (≥4 cut-off score in the range 0–7 for the CITI-SF at T1) and 12.7% at T2 (≥16 cut-off score in the range 0–60 for the CES-D at T2). Averaged across the sample, the levels of subjective sleep quality were poor, with an overall PSQI score of 5.64 on a 0 to 21 possible score range (lower scores representing better sleep, with ≥ 5 cut-off score for sleep disorders). The distribution of the overall PSQI score is presented in S1 Fig. The average amount of sleep (also based on self-reported assessment) was 6.65 hours (*SD* = 1.13). The median sleep efficiency was 82.3% (100% = best sleep efficiency) across 7 days. Most participants (92.8%) reported at least one chronic health condition. The majority of participants were overweight, with an average BMI of 28.90 (*SD* = 5.73), and about one third (34.5%) were taking medications to lower blood pressure.

**Table 1:** Sample characteristics of the final sample (*n* = 968).

	Total	Women (*n* = 525)	Men (*n* = 443)	*p*
Demographics at T1				
Age, y, mean (SD)	46.26 (11.75)	45.79 (11.51)	46.82 (12.03)	.175
Education: ≤ College, n (%)	705 (72.83)	361 (68.76)	344 (77.65)	< .01
Race: White, n (%)	912 (94.21)	492 (93.71)	420 (94.81)	.468
Household income, $, median (IQR)	66250 (38000, 112500)	63000 (34000, 110750)	71500 (41500, 115500)	< .05
Emotional disorders (Depression, anxiety, or others), n (%) ^a^	196 (20.25)	140 (26.77)	56 (12.73)	< .001
Depressive symptoms				
CITI-SF 4 ≥ at T1, n (%)	133 (13.74)	93 (17.71)	40 (9.03)	< .001
CITI-SF, median (IQR), 0–7	0 (0, 0)	0 (0, 0)	0 (0, 0)	< .001
CES-D 16 ≥ at T2, n (%)	123 (12.71)	70 (13.33)	53 (11.96)	.524
CES-D, median (IQR), 0–60	6 (2, 11)	6 (2, 11)	5 (2, 10)	.523
Sleep indicators^b^				
PSQI overall score for sleep quality, mean (SD) at T2	5.64 (3.40)	6.12 (3.60)	5.07 (3.05)	< .001
Sleep duration, mean (SD) at T2	6.65 (1.13)	6.95 (1.12)	6.96 (1.11)	.929
Sleep efficiency (Actigraphy), 0–100, median (IQR) at T3	84.27 (84.27, 88.16)	85.71 (81.53, 89.65)	81.34 (76.14, 86.04)	< .001
Physical health at T2				
Chronic disease, n (%)	898 (92.77)	492 (93.71)	406 (91.65)	.216
Body mass index, mean (SD)	28.90 (5.73)	28.36 (6.12)	29.54 (5.15)	< .01
Smoking, n (%)	426 (44.01)	214 (40.76)	212 (47.86)	< .05
BP meds, n (%)	334 (34.50)	184 (35.05)	150 (33.86)	.699
Cholesterol meds, n (%)	285 (29.44)	122 (23.24)	163 (36.79)	< .001
Inflammatory markers at T2 ^c^				
CRP (mg/L), mean (SD) at T2	2.12 (2.12)	2.38 (2.28)	1.80 (1.87)	< .001
IL-6 (pg/mL), mean (SD) at T2	2.59 (2.48)	2.64 (2.61)	2.56 (2.33)	.869

T1 = baseline, T2 = 11 years from the baseline, T3 = 5 days after T2; IQR = Interquartile range was reported for variables with skewed distribution; CITI-SF ≥ 4 and CES-D ≥ 16 indicates individuals at risk of clinical depression; BP meds = blood pressure medication usage; ^a^ n = 963 due to 5 participants who refused to answer

^b^ *n* = 877 for sleep quality, *n* = 965 for sleep duration, and *n* = 276 for actigraphy assessments were included

^c^ Although natural transformed values were used in all analyses, raw values were reported for interpretation.

Significant gender differences were observed in education, household income, depressive symptoms at baseline, sleep quality and efficiency, BMI, smoking, medication use, and levels of CRP (*p’s* < .05). Compared to men, women had lower education, lower income, were more likely to report any experience or treatment for emotional disorders, were two times more likely to be classified as depressed at baseline, and appeared to have worse subjective sleep quality but better actigraphy-assessed sleep efficiency. Women were also less likely to smoke, or to use medication for high cholesterol, and had a lower BMI but higher CRP levels. Although women reported more depressive symptoms than men at T2, this difference was not significant (*p* = .524).

Bivariate correlations among key study variables and covariates are presented in Table 2. As expected, depressive symptoms at T1 and T2 (11 years later) were moderately correlated (*r* = .21, *p* < .001). Those who exhibited more baseline depressive symptoms were more likely to be younger, women, to earn less income, to report smoking, and to have higher BMI and CRP levels at T2 (*r’s* = from -.10 to .12, *p*’s < .05). Depressive symptoms at T2 were also associated with T2 CRP (*r* = .07, *p* < .05) and IL-6 (*r* = .08, *p* < .05). Depressive symptoms at either time point were associated with worse subjective sleep quality at T2 (T1 symptoms: *r* = .19, T2 symptoms: *r* = .46 both *p’s* < .001) but were not associated with T2 sleep duration or T3 sleep efficiency.

**Table 2:** Correlations among measured indicators (*n* = 968).

	1	2	3	4	5	6	7	8	9	10	11	12	13	14	15
1. Depressive symptoms T1	-														
2. Depressive symptoms T2	**.21** ^**^	-													
3. Sleep quality at T2^a^	**.19** ^**^	**.46** ^**^	-												
4. Sleep efficiency at T3^b^	-.07	-.09	**-.22** ^**^	-											
5. CRP at T2	**.12** ^**^	**.07** ^*^	**.14** ^**^	-.04	-										
6. IL-6 at T2	.05	**.08** ^*^	**.10** ^**^	**-.15** ^**^	**.44** ^**^	-									
7. Age at T1	**-.10** ^**^	**-.13** ^**^	-.04	.08	.04	**.23** ^**^	-								
8. Gender (men) at T1	**.12** ^**^	.04	**.16** ^**^	**.31** ^**^	**13** ^**^	.01	-.04	-							
9. Education (≤ college) at T1	-.01	**-.09** ^**^	-.05	-.10	**-.09** ^**^	-.04	-.05	**-.10** ^**^	-						
10. Race (not White) at T1	.00	-.01	.02	-.01	-.01	.01	.05	-.02	-.01	-					
11. Income^c^ at T1	**-.09** ^**^	-.05	**-.09** ^**^	-.07	**-.09** ^**^	**-.09** ^**^	-.02	-.06	**.20** ^**^	.06	-				
12. Chronic disease at T2	-.04	**-.10** ^**^	**-.17** ^**^	**.12** ^*^	**-10** ^**^	**-.14** ^**^	**-.18** ^**^	-.04	**-.07** ^*^	-.02	-.01	-			
13. Body mass index at T2	**.10** ^**^	**.08** ^**^	**.09** ^*^	**-.13** ^**^	**.42** ^**^	**.32** ^**^	-.01	**-.10** ^**^	-.05	**-.07** ^*^	-.04	**-.12** ^**^	-		
14. Smoking status at T2	**.07** ^*^	**.10** ^**^	.02	.02	.06	**.10** ^**^	**.10** ^**^	**-.07** ^*^	**-.13** ^**^	-.01	-.06	-.06	.02	-	
15. BP meds at T2	-.04	**-.06** ^*^	**-.14** ^**^	.01	**-.12** ^**^	**-.25** ^**^	**-.34** ^**^	-.01	.01	.04	.03	**.20** ^**^	**-.20** ^**^	-.06	-
16. Cholesterol meds at T2	-.02	-.02	.05	.05	.02	**-.16** ^**^	**-.27** ^**^	**.15** ^**^	-.01	-.01	-.04	**.18** ^**^	**-.15** ^**^	**-.09** ^*^	**.32** ^**^

*Note*.

** *p* ≤ 0.01

* *p* ≤ 0.05; Bolded is p *p* ≤ 0.05

^**a**^ Lower scores are indicative of greater sleep quality (*n* = 877)

^**b**^ Higher scores are indicative of greater average actigraphy assessed sleep efficiency across 7 days (*n* = 276)

^**c**^ Due to a non-normal distribution of household income, analyses were conducted using a quintile score. BP meds = blood pressure medication usage.

Sleep indicators were associated with each other, such that subjective sleep quality was associated with objective sleep efficiency (*r* = -.22, *p* < .01). Sleep duration categories of short, normal, and long sleep (*F* (2, 476) = 73.27, *p* < .001) were differently associated with sleep quality. Post-hoc analysis showed that those with short sleep duration (< 7 hours) reported worse overall sleep quality than those with 7–9 hours of sleep (*p* < .001). The correlations between sleep indicators and inflammatory markers showed that lower overall subjective sleep quality was associated with higher IL-6 (*r* = .10, *p* < .01) and CRP (*r* = .14, *p* < .01). Lower objective sleep efficiency at T3 was associated with higher IL-6 (*r* = -.15, *p* < .05). Sleep duration exhibited no associations with inflammation.

### The direct association of baseline depressive symptoms with CRP and IL-6

For all models with CRP, depressive symptoms at T1 were associated with CRP levels at T2 (see Table 3). This association remained significant in the final model, which included all covariates (b = .22, 95% CI = ranged from .10 to .34, *p* < .001). Depressive symptoms at T1 were not significantly associated with IL-6 at T2 in any model in the full sample (as indicated by b = .14, 95% CI = ranged from -.02 to .32, *p* = .10 in the final model). The unadjusted results of the main effects of depressive symptoms on both inflammatory markers were similar to fully adjusted models and are presented in S1 Table.

**Table 3:** Main effects of depressive symptoms at baseline on CRP and IL-6 (*n* = 968).

	*CRP* at T2 *b (95% CI)*			IL-6 at T2 *b (95% CI)*		
	*Model 1*	*Model 2*	*Final model*	*Model 1*	*Model 2*	*Final model*
Depressive symptoms						
T1 symptoms	.22 (.10, .35)***	.22 (.10, .34)***	.22 (.10, .34)***	.14 (-.02, .31)	.14 (.00, .33)	.14 (-.02, .32)
T2 symptoms	.01 (.00, .02)*	.00 (.00, .01)	.00 (-.00, .01)	.01 (.00, .02)**	.00 (.00, .05)	.01 (.00, .01)*
Demographics at T1						
Age	.00 (.00, .01)		.01 (.00, .01)	.01 (.01, .02)***	-	.01 (.01, .01)***
Women	.25 (.12, .38)***		.33 (.21, .45)***	.01 (-.08, .10)	-	.06 (-.01, .15)
Education (≤ College)	-.15 (-.29, .01)*		-.09 (-.21, .06)	.01 (-.10, .11)	-	.03 (-.06, .13)
Race (Not White)	-.04 (-.32, .24)		.10 (-.14, .35)	.02 (-.21, .22)	-	.11 (-.08, .28)
Household income	-.03 (-.08, .03)		-.02 (-.07, .03)	-.05 (-.08, -.01)**	-	-.05 (-.08,-.02)**
Physical health at T2						
Chronic disease		-.21 (-.47, .05)	-.16 (-.42, .12)	-	-.16 (-.34, .01)	-.09 (-.27, .07)
Body mass index		.08 (.07, .09)***	.08 (.07, .09)***	-	.03 (.03, .04)***	.04 (.03, .04)***
Smoking (No smoking)		.10 (-.02, .22)	.10 (-.02, .23)		.10 (.02, .18)*	.08 (.00, .16)
BP meds		-.14 (-.27, .00)*	-.08 (-.21, .07)	-	-.23 (-.32, -.14)***	-.15 (-.24, -.06)**
Cholesterol meds		.26 (.13, .41)***	.22 (.08, .36)**	-	-.07 (-.17, .02)	-.05 (-.14, .05)

*** *p* ≤ 0.001

** *p* ≤ 0.01

* *p* ≤ 0.05; BP meds = blood pressure medication usage; Model 1 includes depressive symptoms at T1 and T2 and demographic covariates; Model 2 adds health covariates to Model 1; b is unstandardized coefficient with 1000 times bootstrapped 95% bias-corrected confidence interval.

### Mediation by sleep quality indicators

**Fig 2:**
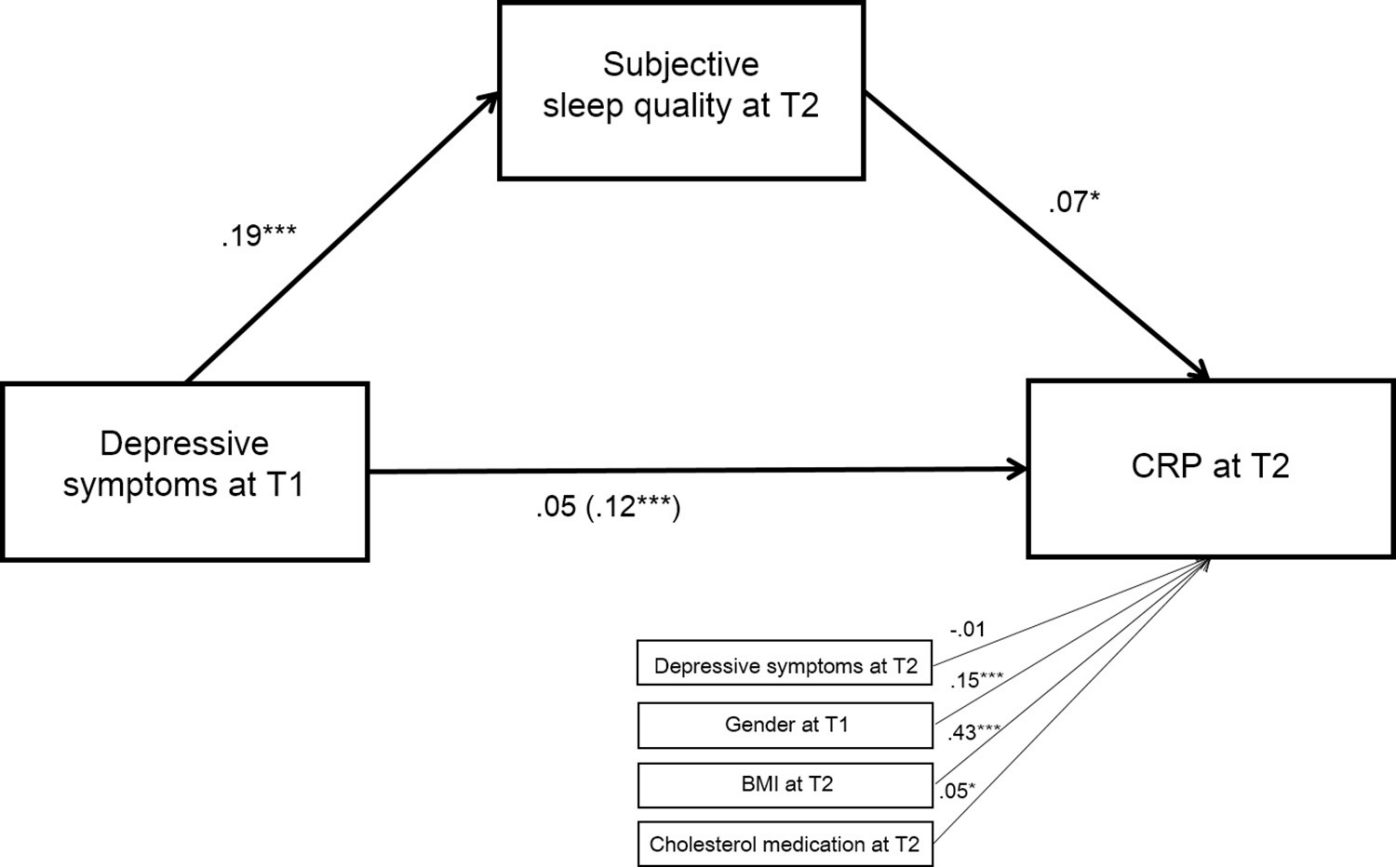
Mediation by subjective sleep quality on the association between depressive symptoms at T1 and CRP at T2. * *p* ≤ .05, ** *p* ≤ .01, *** *p* ≤ .001.The standardized coefficients are reported. The direct effect of depressive symptoms at T1 on CRP at T2 before adding the mediator is presented in parentheses. All marginally significant or significant covariates from the final model for CRP (see Table 3) were included in the model.

As presented in Fig 2, subjective overall sleep quality at T2 significantly mediated the association between depressive symptoms at T1 and CRP levels at T2 (indirect effect; b = .01, 95% CI = .001, .02, *p* ≤ .05) after adjusting for depressive symptoms at T2, gender, BMI, smoking, and medication use for cholesterol (all covariates were at least marginally associated with CRP in the final model in Table 3). Specifically, depressive symptoms at T1 were associated with worse overall sleep quality at T2, which was, in turn, associated with higher levels of CRP at T2. After adding self-reported sleep quality as a mediator, the direct association between T1 depressive symptoms and CRP became non-significant (*p* = .14). Neither sleep duration specifically (examined separately from overall sleep quality) nor objective sleep efficiency mediated the association between T1 depressive symptoms and CRP levels in models with or without covariates (*p* = .47 for sleep duration and *p* = .74 for objective sleep efficiency with covariates). There was no evidence of mediation of the association between T1 depressive symptoms and IL-6 by any of the sleep variables (*p* = .39 for subjective overall sleep quality, *p* = .64 for sleep duration specifically, and *p* = .42 for objective sleep efficiency). The unadjusted results of the mediation effects of sleep quality, duration, and efficiency are presented in S2 Table: in these unadjusted models, overall sleep quality significantly mediated the association between baseline depressive symptoms and both inflammatory markers (CRP and IL-6).

### Moderation by gender

Gender significantly moderated the association between baseline depressive symptoms and IL-6 at T2 (Chi-square difference test: *χ*^*2*^(1) = 5.00, *p* ≤ .05). The unadjusted results were similar to the adjusted results (see S1 Table). As presented in Table 4, a significant association between T1 depressive symptoms and T2 IL-6 was found in women (*b* = .29, 95% CI = .06, .53, *p* < .05) but not in men (*b* = -.05, 95% CI = -.26, .15, *p* = .654) after adjusting for all final model covariates. Gender did not moderate the association between T1 depressive symptoms and T2 CRP (*χ*^*2*^*(1)* = .50, *p* = .48); in men, this association was marginally significant (*b* = .15, 95% CI = .00, .30, *p* = .06), whereas in women it was significant (*b* = .23, 95% CI = .07, .39, *p* < .01). Finally, gender did not moderate any of the mediation models. We subsequently explored whether menopausal status or any use of hormone therapy explained the gender differences in IL-6: The significant association between T1 depressive symptoms and T2 IL-6 in women was not affected by adjusting for menopausal status (*b* = .37, *95% CI* = .06, .71, *p* = .03; *n* = 337) or use of hormone therapy (*b* = .37, *95% CI* = .04, .72, *p* = .03; *n* = 345).

**Table 4:** Gender moderation of the final model for effects of depressive symptoms at baseline on inflammatory markers (*N* = 968).

	CRP at T2 *b (95% CI)*		IL-6 at T2 *b (95% CI)*	
	Women	Men	Women	Men
Depressive symptoms				
Depressive symptoms at T1	.23(.07 .39)**	.15 (.00, .30)†	.29 (.06, .53)*	-.05 (-.26,. 15)
Depressive symptoms at T2	.00 (-.01, .01)	.01 (-.01, .02)	.16 (-.34, .58)	.47 (.11,. 91)*
Demographics				
Age	.00 (-.01 .01)	.01 (.00, .01)	1.08 (.38, 1.83)**	1.95 (1.16, 2.79)***
Education (Some college)	-.06 (-.24, .12)	-.16 (-.38,.05)	.01 (-.02, .03)	.01 (-.02, .03)
Race (Not White)	.08 (-.27, .38)	.17 (-.25, .54)	.00 (-.01, .01)	.01 (.00, .03)
Household income	-.03 (-.10.04)	-.01 (-.08, .07)	-.11 (-.19, -.03)*	-.06 (-.14, .02)
Physical health				
Chronic health conditions	-.37(-.75, .03)*	.04 (-.35, .43)	.00 (-.01, .01)	-.01 (-.03, .01)
Body mass index	.09(.07, .10)**	.07 (.05, .09)***	1.53 (1.12, 1.92)***	.77 (.50, 1.08)***
Smoking (No smoking)	-.04 (-.21, .12)	.26 (.07, .43)**	.02 (-.01, .05)	.01 (-.02, .05)
BP meds	-.05 (-.22, .13)	-.07 (-.28, .17)	-.03 (-.06, .00)*	-.03 (-.06, -.01)*
Cholesterol meds	.27 (.09, .46)**	.14 (-.08, .36)	.00 (-.02, .02)	-.02 (-.05, .01)
Χ^2^ difference test	*Χ^2^*(1) = .50, *p* = .48		*Χ^2^*(1) = 3.81, *p* = .05	

*** *p* ≤ 0.001

** *p* ≤ 0.01

* *p* ≤ 0.05

† = *p* = .06; BP meds = blood pressure medication usage; b is unstandardized coefficient with 1000 times bootstrapped 95% bias-corrected confidence interval.

## Discussion

Using a national probability cohort of middle-aged adults in the United States, we examined the long-term effects of depressive symptoms on inflammation (via levels of CRP and IL-6), as well as whether two key indicators of sleep disturbances (overall subjective sleep quality and actigraphy-assessed sleep efficiency) can help to explain such associations. Baseline depressive symptoms were associated with levels of inflammation 11 years later; this was seen for CRP in the full sample with both men and women, and for IL-6 in women only, even after adjusting for concurrent depressive symptoms, age, SES, race, BMI, smoking, chronic health conditions, and medication use. In addition, self-reported overall sleep quality accounted for the association between depressive symptoms at baseline and later CRP levels. These findings advance the literature on the link between depression and inflammation by 1) showing gender-specific associations across time in a non-clinical sample of adults, and 2) identifying impaired quality of sleep as one of the potential pathways to explain this linkage.

### Depression is associated with inflammatory markers

The current study observed an apparent long-term association between depressive symptoms and CRP in the full sample with both men and women, and between depressive symptoms and IL-6 in women (over and above concurrent depressive symptoms, relevant demographic characteristics and health status indicators). It is important to note that effect sizes observed in the present research were small (e.g., r = .12 for the association between baseline depressive symptoms and CRP); such effect sizes are consistent with previous findings. Smaller effect size have been found between self-reported depressive symptoms and inflammation in community based samples relative to more modest effect sizes when using clinical assessments of depression in patient samples [14,58]. This said, the magnitude of the associations observed in the present research may have clinical significance. In the present sample, for the main effect of depressive symptoms on CRP, each additional depressive symptom was associated with a 1.25 mg/L higher levels in CRP for both genders and a 1.40 pg/mL higher levels in IL-6 for women. This indicates that the experience of even mild depressive symptoms has the potential to be associated with higher systemic inflammation a decade later, and in ways that may indicate risk for cardiovascular disease and other inflammatory conditions [62].

This finding extends a 2020 meta-analysis of 107 cross-sectional studies, which reported that both CRP and IL-6 were elevated in depressed patients overall, after taking into consideration a number of lifestyle, biological, and methodological factors [63]. Previous prospective studies generally suggest that depressive symptoms or episodes of depression are associated with CRP levels longitudinally, although there has been some variability in these results. In line with the present findings, a study of individuals who were monitored yearly during adolescence from age 11 to 16 years, and then again at 19 and 21, reported (separate) positive relationships between either depressive symptoms or recurrent episodes of depression at a given visit and CRP at the next visit [27]; notably, only recurrent, cumulative depressive episodes were associated with CRP after accounting for covariates in that study. In the present research we did not have data available on recurrent episodes of depression; however, in this population cohort sample as a whole we observed that levels of depressive symptoms at one time point were associated with CRP (but not IL-6) 11 years later, even after accounting for concurrent depressive symptoms and other covariates. The “scar hypothesis” suggests that impairments in self-image and increased susceptibility to emotional and physiological reactivity to stress may help explain the potentially long-lasting deleterious impact of depressive symptoms on health [32]. Such changes in self-image and stress reactivity may be associated with later health decline by increasing vulnerability to recurring depressive symptoms [64,65], maladaptive health behaviors such as physical inactivity and smoking [66,67], and development of inflammation-related chronic disease conditions [8,68]. Future longitudinal studies are needed to identify the specific biological, psychological, and social processes by which depressive symptoms are linked to later negative health consequences.

In two other studies, depressive symptoms were associated with future CRP levels but only in a subset of participants–i.e., in men but not women [30] or in Black but not White participants [29]. Further, other studies reported null findings when testing relationships between baseline depressive symptoms and CRP one year later in a sample of middle-aged women [31], six years later in a smaller sample (*N* = 263) of older adults [28], and 12 years later in adults from the British civil servant cohort [23]. In the present study, although the association between baseline depressive symptoms and CRP levels 11 years later was marginally significant for men, similar associations with CRP were observed in both men and women; it should be noted that most of our participants were White. One potential explanation for such discrepancies between studies pertains to demographic differences between studies, as the pattern of association between depression and inflammatory markers may vary based on the individual characteristics of a given sample, including in terms of stress exposure, gender, race, or other demographics [69]. Alternatively, there may be differences between studies in the manner by which different depression scales assessed somatic symptoms of depression. For example, the depression measure in the present study included somatic symptoms, whereas the measure in the Gimeno study (with null results) assessed only cognitive symptoms of depression. Importantly, both Niles’ and Deverts’ groups note that their findings with CRP were mostly driven by somatic symptoms of depression. The association between depression and inflammation may be particularly robust when somatic symptoms of depression are included [41]. Future studies are needed, including those that include a stratified sampling strategy for examining detailed subgroup differences by which somatic, cognitive, and affective symptoms of depression could be examined strategically.

### Gender moderation of the association between depressive symptoms and inflammation

Whereas depressive symptoms were associated with CRP in both women and men in the present research, they were associated with IL-6 only among women. This gender difference was not explained by the two potential mechanisms we were able to test: women’s menopausal status or use of hormonal therapy. Previous studies have been mixed with regard to gender differences in the association between depression and inflammation. For example, one prospective study found positive associations between depressive symptoms and CRP only in men [30], and two prospective studies did not find that gender moderated associations between depression and CRP or IL-6 [28,70]. Further, in two cross-sectional studies, only women showed an association between depressive symptoms and inflammatory markers–CRP in one study [71] and TNF-α in another [72]. Such findings illustrate the importance of examining gender as a moderator, as opposed to only treating it as a covariate. As noted in the previous section, inflammation may be related to specific dimensions or symptoms of depression and the pattern of these specific associations may further differ across gender. Future prospective studies are needed to clarify generalizability of the present findings and the specific biological or psychosocial mechanisms that underlie such gender specific patterns.

### Role of sleep

In the present research, self-reported overall sleep quality but not actigraphy-assessed sleep efficiency mediated the longitudinal association between depressive symptoms and CRP. Sensitivity analyses, with sleep duration specifically, suggested that the impact of overall sleep quality in this study was not explained by sleep duration. To our knowledge, this is the first study to show that the association between depressive symptoms and inflammatory markers can be driven by impaired sleep quality. These results are in line with findings from the Netherlands Study of Depression and Anxiety, which showed that health behaviors explained the longitudinal association between depressive symptoms and higher levels of CRP and IL-6 [66], although that study focused on smoking and physical activity and did not investigate the role of sleep. Our results are also consistent with extant literature demonstrating a strong (bidirectional) link between depression and sleep disorders [73] and that poor sleep quality is associated with elevated inflammation [47]. In line with a recent finding suggesting that sleep intervention can help decrease levels of depressive symptoms and inflammation [74], the present findings suggest there may be potential benefits of sleep quality management in preventing systemic inflammation among those with depressive symptoms. One mechanistic explanation integrating the previous literature and current findings posits that poor sleep quality among individuals with depression can cause fatigue, and that both symptoms may lead to a more sedentary lifestyle and/or unhealthy behaviors, which in turn may result in elevated inflammation [41]. It is important to note that there may be alternative relationships among depression, sleep, and inflammation that were not modeled in the present study. For example, early adversity or childhood exposure to inflammatory conditions might interact or prime individuals toward worse sleep or depression in ways that could not be tested in the present work. Moreover, due to the bidirectional nature of the associations between sleep and inflammation [47,75] inflammatory markers may instead play a mediating role in the association between depressive symptoms and sleep disturbances; that is, depressive symptoms may trigger greater production of inflammatory cytokines (e.g., IL-6 and TNF), which may subsequently impair sleep [18]. Future studies with assessments of depressive symptoms, sleep indicators, and inflammatory markers at multiple time points are needed to test this and other possible explanations for how depression, sleep, and inflammation are connected over time.

The observed inconsistency among different measures of sleep in the present study is relatively common in the sleep literature [76,77] and likely reflects that different measures reflect distinctive but interconnected aspects of sleep health [78]. Two studies in older adults 65+ years reported associations between depression status and subjective sleep quality/disruption, but not with objective sleep efficiency [77,79]. Interestingly, in a study of middle-aged adults, there was less concordance between perceived sleep quality and self-reported sleep duration among individuals with higher depressive symptoms [76]. A potential explanation for a disconnect between perceived sleep quality and other measures of sleep may be that negative attention biases related to depression lead to over-reporting or exaggerated estimations of poor sleep quality [80]. It is also possible that actigraphy-assessed sleep efficiency fails to capture meaningful aspects of the sleep experience, such as undetected brief episodes of wakefulness that may affect how rested participants feel [81].

The present results with sleep can also be compared to several past studies that have focused on a linkage between sleep indicators and inflammatory markers in other contexts [47]. A meta-analysis of cross-sectional studies on sleep and inflammation reported that both poor subjective sleep quality and longer sleep duration (>8 hours) were associated with higher CRP and IL-6, whereas shorter sleep duration (<7 hours) was related to higher IL-6 but not CRP in populations without diagnosed sleep disorders (Irwin et al., 2016). The current results partially support these findings by showing that lower overall subjective sleep quality was associated with higher concurrent levels of IL-6 and CRP, whereas, when examined separately, the sleep duration indicators were not. However, the present study did not observe a significant association between 7-day assessment of sleep efficiency in either IL-6 or CRP; in contrast, a study of midlife women found that 3-day assessment of poorer objective sleep efficiency was related to higher IL-6 levels, albeit not CRP [82]. An important target for future research will be to clarify specific methodological differences in the use of different sleep indicators or sample characteristics that may relate to discrepancies across such studies.

### Limitations and future directions

Although this study adds new evidence that impaired sleep quality may be one of the factors that explain a longitudinal association between depression and inflammation, several factors should be taken into consideration when interpreting these results. First, we were unable to control for baseline levels of inflammation or examine changes in inflammation across time, as inflammatory markers were only assessed at T2. As emerging evidence suggests that there are bi-directional associations between both inflammation and depression as well as between sleep and depression [83,84], future studies with additional time points of inflammation and sleep assessments are needed to further tease apart these temporal associations. Additionally, subjective sleep quality and sleep duration over the last month were reported at T2 (i.e., the same time as measures of inflammation) whereas actigraphy-assessed sleep efficiency was measured 5 days after T2, which was not ideal for a clear understanding of directionality between sleep and inflammation using mediational analysis. It is important to remind readers that analyses involving measures of sleep quality and duration were statistically redundant because sleep quality was calculated by the overall PSQI score, which included a sleep duration as one of the items; thus, sleep duration was used as a sensitivity analysis in the present study, to determine whether the significant mediation by overall sleep quality was driven or affected by abnormal sleep duration. A strength of our analytical approach was that we controlled for depressive symptoms at T2 (concurrent with inflammatory markers and sleep measures), utilized data from a large national probability cohort sample, and applied both self-reported and objective measures (using actigraphy) of sleep disturbances.

As with much of the prior literature, depressive symptoms in this study were relatively mild. Distinct episodes of clinical depression over the years may play a cumulative role, as suggested by Copeland and colleagues [27], but this could not be examined in this study. It is also noteworthy that baseline symptoms of depressive symptoms were associated with later symptoms only modestly (albeit significantly) (r = .21), which is consistent with a review of 18 adult population cohort studies that showed instability in long-term trajectories of depressive symptoms in adults [for review, see 85]. Additionally, although the CIDI-SF used in MIDUS captures a number of different types of depressive symptoms, participants were not asked about other depressive symptoms if they did not report feeling “sad, blue, or depressed” almost every day for at least 2 weeks over the last year. This may have limited detection of depressive symptomatology, some of which (such as somatic symptoms) may be more strongly associated with inflammation.

Sample characteristics limit generalizability and may have affected results. Although the study sample appeared to be broadly similar to U.S. midlife adults in terms of rates of emotional disorders [86], obesity [87] and chronic disease conditions such as hypertension [88], participant attrition (including due to death) at T2 and inclusion based on proximity to a testing center at T3 may have affected results. Further, our sample consisted of predominantly well-educated, White Americans. Future research is needed to better understand the role of physiological and contextual factors that may help explain gender differences in the context of depression and inflammation. Other factors that can vary between men and women–including biological factors, such as the levels of endotoxin in the blood [89], or social factors, such as the greater impact of depressive symptoms on social stress and loneliness among women [17]–may be particularly relevant.

## Conclusion

Depressive symptoms were associated with inflammation 11 years later in a nationally representative sample of adults. Notably, the present results show that depressive symptoms were linked with inflammation above and beyond concurrent depression as well as current health conditions and demographic factors. Results suggest that women may evidence a more consistent pattern of associations between depressive symptoms and both inflammatory markers examined (CRP and IL-6) compared to men (in whom marginal associations were only observed with CRP). Finally, results showing that overall sleep quality explained the connection between baseline depressive symptoms and later inflammation suggest that intervening on subjective feelings of disturbed sleep may help mitigate the negative health consequences of depressive symptoms. To elucidate such novel points of intervention, it would be valuable to invest in future research to further untangle the temporal unfolding of depression, sleep disturbances, and inflammation, as well as the biopsychosocial context underlying gender differences in these dynamics.

## Supporting information

S1 Fig Distribution of the overall PSQI score in the study sample (*n* = 968). (TIF)

S1 Table Unadjusted results of main effects of depressive symptoms at baseline on CRP and IL-6 in total sample and by gender (*n* = 968). (PDF)

S2 Table Unadjusted results of mediation effects of sleep quality, duration, and efficiency on the association between depressive symptoms at baseline on CRP and IL-6. (PDF)
