# Supplementary figures and images for: The longitudinal connection between depressive symptoms and inflammation: Mediation by sleep quality
Source: PLoS One. 2022 May 26;17(5):e0269033. doi: 10.1371/journal.pone.0269033 (PMC9135207; doi:10.1371/journal.pone.0269033)

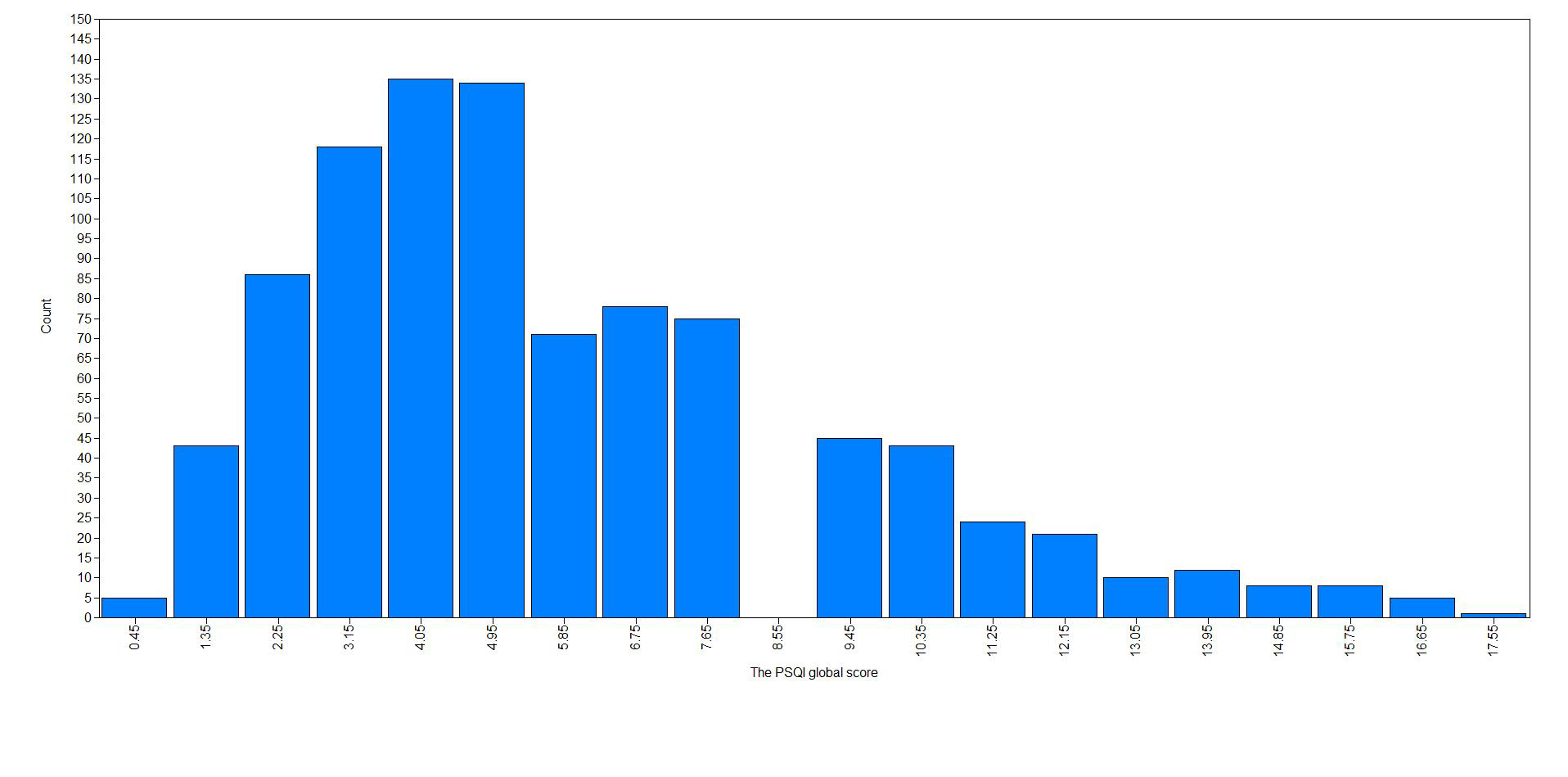

Supplement: S1 Fig — (TIF) [file pone.0269033.s001.tif]
