# Supplementary material for: The longitudinal connection between depressive symptoms and inflammation: Mediation by sleep quality
Source: PLoS One. 2022 May 26;17(5):e0269033. doi: 10.1371/journal.pone.0269033 (PMC9135207; doi:10.1371/journal.pone.0269033)
Supplement: S1 Table — (PDF) [file pone.0269033.s002.pdf]

Supplemental Table 1. Unadjusted results of main effects of depressive symptoms at baseline on CRP and IL-6 in total sample and by gender (*N* = 968)

	CRP at T2 *b (95% CI)*			IL-6 at T2 *b (95% CI)*		
	Total	*Women*	*Men*	*Total*	*Women*	*Men*
Depressive symptoms	.219 (.109, .341)***	.229 (.036, .402)**	.149 (-.001, .314)	.142 (-.011, .318)	.288 (.067, .508)**	-.046 (-.254, .157)

*** $p \leq 0.001$, ** $p \leq 0.01$, * $p \leq 0.05$; b is unstandardized coefficient with 1000 times bootstrapped 95% bias-corrected confidence interval.
