# Supplementary material for: The longitudinal connection between depressive symptoms and inflammation: Mediation by sleep quality
Source: PLoS One. 2022 May 26;17(5):e0269033. doi: 10.1371/journal.pone.0269033 (PMC9135207; doi:10.1371/journal.pone.0269033)
Supplement: S2 Table — (PDF) [file pone.0269033.s003.pdf]

Supplemental Table 2. Unadjusted results of mediation effects of sleep quality, duration, and efficiency on the association between depressive symptoms at baseline on CRP and IL-6

Mediators	Indirect effects *b (95% CI)*	
	Depression at T1 → *CRP* at T2	Depression at T1 → IL-6 at T2
PSQI global sleep quality	.012 (.005, .022)**	.006 (.002, .012)*
Sleep duration	.001 (-.001, .007)	.000 (.000, .003)
Sleep efficiency	.001 (-.003, .013)	.004 (-.002, .015)

*** $p \leq 0.001$, ** $p \leq 0.01$, * $p \leq 0.05$; b is unstandardized coefficient with 1000 times bootstrapped 95% bias-corrected confidence interval; The indirect effect by each of the three sleep mediators were reported as a measure of mediation.
